# Seroprevalence of *Peste des Petits* Ruminants and Contagious Caprine Pleuropneumonia Coinfections in Goats in Kwale County, Kenya

**DOI:** 10.1155/2023/5513916

**Published:** 2023-07-14

**Authors:** George Lugonzo, George Gitao, Lilly Bebora, Harrison Osundwa Lutta

**Affiliations:** ^1^Kenya Agricultural and Livestock Research Organization, Biotechnology Research Institute, P.O. Box 14733-00800, Nairobi, Kenya; ^2^University of Nairobi, Department of Veterinary Pathology, Microbiology and Parasitology, Nairobi, Kenya

## Abstract

Goats are among the most important small ruminants affected by *Peste des Petits* ruminants (PPR) and contagious caprine pleuropneumonia (CCPP) diseases, two of the most significant constraints worldwide to the production of small ruminant species. Herein, the competitive enzyme-linked immunosorbent assay (cELISA) and the latex agglutination test (LAT) were used to determine the coinfections of PPR and CCPP in goats in Kwale County on Kenya's South Coast. A total of 368 serum samples were collected from goats of various ages and sexes exhibiting respiratory distress in the four subcounties of Kwale County (Kinango, Lunga Lunga, Matuga, and Msambweni) and screened for PPR and CCPP antibodies. Of the 368 goats sampled, 259 (70.4%) were females and 109 (29.6%) were males, and 126 (34.2%), 71 (19.3%), 108 (29.3%), and 63 (17.1%) samples were collected from Kinango, Matuga, Lunga Lunga, and Msambweni, respectively. The overall PPR seropositivity rate was 48.6% (179/368); rates in Kinango, Lunga Lunga, Matuga, and Msambweni were 70.6%, 29.6%, 49.3%, and 36.5%, respectively. The overall CCPP seropositivity rate was 45.4% (167/368), while rates in Kinango, Lunga Lunga, Matuga, and Msambweni were 51.6%, 49.1%, 36.6%, and 36.5%, respectively. Notably, the seropositivity of PPR was higher in male (53.3%) than in female (46.72%) goats, though not statistically significant. In addition, the CCPP seropositivity rates were not significantly different between male (44.0%) and female (45.9%) goats. Regarding age, the PPR seropositivity rates were 45.9%, 55.8%, and 52.3% in adults, kids, and weaners, respectively. For CCPP, the seropositivity rates were 48.3%, 40.4%, and 42.3% in adults, kids, and weaners, respectively. The coinfection rate of PPR and CCPP was 22.3% (82/368). Despite the high coinfection, univariate analysis revealed no relationship between PPR and CCPP infections. However, given the high PPR and CCPP infection rates, as a result of separate or coinfection, there is a need to upscale or intensify vaccination in the county.

## 1. Introduction

Small ruminants are critical livestock production units worldwide, particularly in underdeveloped nations [1], where they are reared for income, food [2], and social functions [3]. Optimal goat production is threatened by numerous limitations, including inadequate nutrition, poor breeds, poor management programs, and various diseases. Infectious diseases are a serious constraint to animal production globally [4]. Diseases result in direct and indirect losses, including death, increased production costs, decreased productivity, and trade restrictions [5]. PPR is one of the most severe contagious respiratory viral illnesses affecting goat production in developing countries [6], characterized by bronchointerstitial pneumonia, fever, fibrinonecrotic tracheitis, necrotic-ulcerative stomatitis, diarrhea, and mouth sores, with significant variations between individual animals and locations [7, 8]. PPR is caused by the *Peste des Petits* ruminant virus (PPRV), a morbillivirus in the Paramyxoviridae family. Direct contact between infected and susceptible animals is the main mode of transmission of the virus. The morbidity and mortality rates associated with PPR depend on numerous factors, including the species and age of the animal, underlying infections, and the host's resistance [9, 10]. The morbidity rate of PPR can reach 100%, underlining the impact of the disease on the livelihoods of livestock keepers and the small ruminant trade [11].

CCPP is another highly contagious respiratory disease of goats caused by *Mycoplasma capricolum* subspecies *capripneumoniae* (Mccp), posing a severe threat to goat populations [12]. The disease causes heavy economic losses in low and middle-income nations relying on goat farming, especially in Africa and Asia [13], and these losses are similar to those caused by PPR [14, 15]. Morbidity and mortality rates due to CCPP in exotic breeds can be as high as 100% [16] and up to 80% in disease-naive local breeds. In endemic areas, the total annual cost of CCPP is estimated to be around US$507 million, underlining the substantial losses caused by the disease. Clinically, acute infection is characterized by high fever (40.5–41.5 degrees Celsius), anorexia, depression, reluctance to walk, dyspnea, labored breathing, coughing, grunting, and death within five days of the onset of symptoms [17].

Reports of PPR and CCPP prevalence in Kenya remain scanty, despite Kenya being among the countries practicing traditional and extensive goat husbandry. Kenya has adopted a systematic vaccination program, or vaccination is performed following a risk-based analysis to reduce the incidences with a view to prevent or control the disease. The vaccine used against CCPP is CAPRIVAX™, an inactivated vaccine prepared from Mccp, originally known as the F38 biotype. For PPR, the N 75/1 (LK6 Vero 74) vaccine is used, which is a live-attenuated vaccine. Besides regular vaccinations against the PPR and CCPP diseases, frequent outbreaks continue to be observed, particularly in arid and semiarid areas of the country. This study aimed at providing an insight into the seropositivity and the relationship between PPR and CCPP infections in goats in Kwale County, one of Kenya's semiarid regions. The findings of this research will create a rationale for making sound decisions in establishing specific control strategies against the PPR and CCPP diseases to improve goat productivity and, thus, contribute to PPR and/or CCPP eradication by 2030.

## 2. Materials and Methods

### 2.1. Study Area

**Figure 1:**
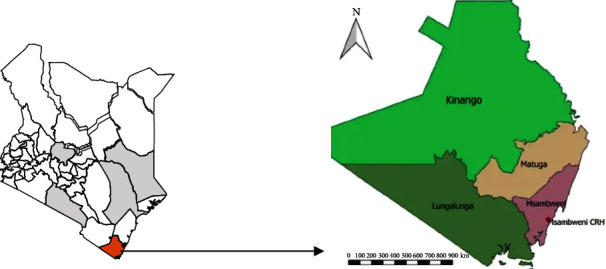
Map of Kenya showing the location of Kwale County.

Kwale County, with a total land area of 8,270.2 square km, is located on the Southern Coast of Kenya and borders Tanzania to the southwest (Figure 1). The 2019 population census estimated the county's human population to be 858,748 [18]. The county lies between latitude 4°10′ 25.50″S and longitude 39°27′7.42″E.

### 2.2. Study Design and Sample Size

Goats with respiratory distress were sampled from the four subcounties of Kwale County (Matuga, Msambweni, Kinango, and Lunga Lunga). The subcounties formed the first sampling units, with wards forming the second sampling unit. Animals were then sampled from villages in the wards. Since there are no data on the seroprevalence of PPR and CCPP in Kwale County, the sample size was calculated using an estimated PPR-CCPP coprevalence of 29.25%, a *Z* value of 1.96 (at 95% CI), and a precision of 5%. 29.25% was obtained from a pilot study of 106 samples conducted in March 2021. Based on the formula *n* = *Z*^2^ *P*(1 − *P*)/(*d*^2^) [19], where *n* is the sample size, *Z* is the statistic corresponding to the level of confidence, *P* is the expected prevalence (obtained from the same studies or a pilot study), and *d* is the precision (corresponding to the effect size), the sample size was calculated as *n* = 1.96^2^ × 0.2925 (1 − 0.2925)/(0.05^2^), giving a total of 318 goats.

### 2.3. Sample Collection, Handling, and Transportation

Blood (10 ml) for screening of PPR and CCPP antibodies was drawn from the jugular vein of goats into non-heparinized vacutainer tubes after cleaning the injection sites with 70% ethanol. The samples were stored in cooler boxes in the field and transported to the Kilifi veterinary laboratories for temporary storage. Sera were extracted from the blood samples after overnight storage at +4°C and stored in 2 ml cryovials. The samples were then transported to the Kenya Agricultural and Livestock Research Organization (KALRO)-Biotechnology Research Centre (BioRC), Kabete, to screen for PPR and CCPP antibodies. Only unvaccinated goats were sampled. The animals sampled for the study had not been vaccinated for at least a year, and the history of vaccination was obtained from the respective farmers. Newly bought animals were not sampled because of unavailability or unreliable vaccination history.

### 2.4. Screening for PPR Antibodies

The screening of anti-PPR antibodies in the serum samples was performed by using a competitive ELISA (cELISA) kit purchased from IDeVet Company Ltd (Grabels, France), following the manufacturer's instructions. The optical densities (ODs) were then obtained at 450 nm using an ELISA reader from Awareness Technologies Ltd. (Palm City, FL, USA).

### 2.5. Screening of CCPP Antibodies Using the Latex Agglutination Test

#### 2.5.1. *Mycoplasma* Culture and Confirmation Using PCR

Mccp culture was obtained from the Kenya Veterinary Vaccines Production Institute (KEVEVAPI) and confirmed using PCR. For the PCR process, 20 ml of the bacterial culture at the logarithmic growth phase (1–3 × 10^10^ cells) was transferred into a 50 ml centrifuge tube and centrifuged at 4,100 rpm for 1 hour at 4°C. The supernatant was discarded, and the pellet was washed three times using 5 ml TES buffer (10 mM Tris-HCl, pH 7.5, 140 mM NaCl, and 1 mM EDTA) at 4,000 rpm for 20 minutes at 4°C. The pellet was resuspended in 100 *μ*L of TE buffer (10 mM Tris-HCl, pH 7.5, 1 mM EDTA, and pH 8.0) and transferred to a 2 ml centrifugation tube. Glycine, NaCl, and EDTA (GES) buffer (500 *μ*L) were added and mixed thoroughly by vortexing. The mixture was incubated for 5–10 minutes at room temperature (a clear lysate forms) and cooled on ice for 3–5 minutes before adding 250 *μ*L of ice-cold 7.5 M NH_4_OAc at a pH of 7.7, mixed by inverting the tubes several times, and incubated on ice for 10 minutes. PCIA (phenol : CHCl_3_ : isoamyl alcohol = 49.5 : 49.5 : 1; 500 *μ*L) was then added, and the mixture was centrifuged at 14,000 rpm for 15 minutes at 4°C. The aqueous phase was transferred into a new Eppendorf tube, and the PCIA process was repeated three times. Isopropanol was added to the solution and mixed well (850 *μ*L of the aqueous phase added to 600 *μ*L of isopropanol). The solution was then incubated at −20°C for at least 20 minutes before centrifugation at 14,000 rpm for 15 minutes at 4°C. The supernatant was discarded, and the pellet was washed three times at −20°C in 80% EtOH (without mixing). Ethanol (800 *μ*L of 80% ethanol) was added and centrifuged at 14,000 rpm for 15 minutes at 4°C. The supernatant was discarded, and the DNA pellets were dried at room temperature for 3 hours in the biosafety cabinet before resuspension in 100 *μ*L of TE (pH 8.0). The DNA concentration was measured using Nanodrop/QUBIT and stored at −20°C until further analysis. The 316 bp *arcD* gene was amplified using 5′-ATCATTTTTAATCCCTTCAAG-3′ and 5′-TACTATGAGTAATTATAATA-TATGCAA-3′ forward and reverse primers, respectively, as previously described by Woubit et al. [20]. The gel electrophoresis results after DNA amplification are shown in Supplementary Figure 1.

#### 2.5.2. Coating of Polystyrene Beads

Polystyrene latex beads (1.08 *µ*l) for the latex agglutination test (LAT) were purchased from Sigma Aldrich Company (St. Louis, Missouri, USA). The F38 polysaccharide of Mccp, mainly consisting of four neutral sugars (glucose, galactose, mannose, and fructose) and two amino acid sugars (glucosamine and galactosamine), was extracted from the Mccp culture as previously described by Rurangirwa et al. [21]. In brief, the pH of the Mccp culture supernatant was adjusted to 5.0 with glacial acetic acid before boiling for one hour and filtering through a Whatman coarse filter paper. The filtrate was mixed thoroughly with two volumes of ethyl-alcohol and then incubated at 4°C for 24 hours. The mixture was centrifuged at 1000g for 15 minutes, and the resultant supernatant was suspended in distilled water and stirred for 2 hours at 37°C. Thereafter, the mixture was span at 3000 g for 30 minutes at 4°C. The supernatant was collected for subsequent processes. An equal volume of aqueous phenol (6 g of phenol in 1.0 ml of distilled water) was added, and the mixture was incubated at 68°C for 1 hour in a water bath. The mixture was then incubated at 4°C overnight before centrifugation at 3000 g for 30 minutes at 4°C. The aqueous phase was separated and dialyzed for two days in running tap water to eliminate all the phenol. Two volumes of ethyl alcohol were added to the dialysate, mixed thoroughly, and incubated overnight at 4°C to precipitate carbohydrate. The mixture was centrifuged at 100g for 15 minutes to fully precipitate the carbohydrate, which was suspended in a small volume of distilled water. The suspension was dialyzed further for two days with at least four changes of water per day. Finally, the carbohydrate was precipitated with two volumes of ethyl alcohol and dissolved in 50 ml of distilled water. The dialysate was aliquoted and stored at −20°C until further analysis.

The concentration of the polysaccharide was determined using the sulphuric acid method. Glucose was used as the standard. All the reagents used were of analytical grade. Several dilutions of the extracted polysaccharide were prepared alongside D-glucose standards of known concentrations (20, 40, 60, 80, 100, 120, 140, 160, 180, and 200 *µ*g/ml). Phenol solution was added to 0.5 ml of the D-glucose solution of each concentration in clean labeled test tubes, mixed, and prechilled on ice. Concentrated sulphuric acid (2.5 ml) was added, followed by quick vortexing. The tubes were then incubated immediately at 68°C for 15 minutes in a water bath. Test samples and water were treated equally. The tubes were cooled at room temperature, and optical densities were determined at 490 nm using an ELISA reader (MR 400 microtiter plate reader, Dynatech instruments, Torrance, CA). The concentration of the polysaccharide was determined using a standard calibration curve drawn from D-glucose readings generated automatically using an ELISA reader. The polysaccharide concentration was adjusted to 1 mg/ml and stored at −20°C. To sensitize the beads, 1 ml of latex beads (10%, 1.08 *µ*m) (St. Louis, Missouri, USA) was incubated with 1 mg/ml of polysaccharide in 1 ml phosphate buffered saline supplemented with 0.2% sodium ethylenediaminetetraacetate and 0.01% sodium azide. The mixture was shaken well and incubated at 37°C for 1 hour before adding 8 ml of the buffer.

#### 2.5.3. Latex Agglutination Test (LAT)

CCPP antibodies were screened using the LAT as described by Rurangirwa et al. [21]. Approximately 10 *µ*l of serum was mixed with an equal volume of polysaccharide-sensitized latex beads on a slide, swirled for 1 minute, and read visually within 1−2 minutes. The development of agglutination, as shown in Supplementary Figure 2, was taken as a positive reaction. The polysaccharide preparation was vortexed every 5 minutes before use.

## 3. Data Analysis

The protocol for this study was approved by the University of Nairobi Faculty of veterinary medicine. Data were analyzed using SPSS software, version 25. Grouped data, including the disease rate and the distribution of goats of different ages, were summarized into percentages. Differences in PPR and CCPP infection rates among kids, weaners, and adults were analyzed using analysis of variance (ANOVA). The relationship between age and sex, and PPR as well as CCPP infection was investigated using univariate logistic regression analysis. Statistical significance was set at *P* < 0.05.

## 4. Results

A total of 368 serum samples from goats in Kwale County were screened for both PPR and CCPP antibodies. Of these, 259 (70.4%) were from females and 109 (29.6%) were from males. Regarding subcounties, 126 (34.2%), 71 (19.3%), 108 (29.3%), and 63(17.1%) were sampled from Kinango, Matuga, Lunga Lunga, and Msambweni, respectively. The serological results are shown in Tables 1 and 2. Kinango had the highest seroprevalence for both PPR and CCPP; Msambweni had the lowest for the two diseases. A proportion of 22.3% of the tested goats were seropositive for both PPR and CCPP (Table 2), indicating coinfection by the two diseases.

### 4.1. The Relationship between PPR and CCPP Infection

Spearman's correlation coefficient analysis found no interdependence between the occurrence of PPR and CCPP infections in goats (*r* = 0.0084, *P*=0.8724). Univariate logistic regression analyses for associations between sex, age, and PPR/CCPP diseases indicated no correlation between the sex and age of the goats and either of the two diseases (Tables 3 and 4).

## 5. Discussion

*Peste des petits* ruminants, a disease primarily affecting small livestock, is gaining recognition globally, but particularly in Africa and Asia. While it is believed that the disease was first introduced in Kenya in 1992 [22], the first officially documented cases in Turkana, Kenya, occurred in 2007 [23]. CCPP is another important disease that constrains goat farming globally, particularly in Asia, Africa, the Middle East, and India. In Kenya, however, reports on the joint prevalence of PPR and CCPP remain scanty, despite Kenya being among the countries practicing traditional and extensive goat husbandry. Besides regular vaccinations against PPR and CCPP, frequent outbreaks continue to be observed, particularly in arid and semiarid areas of the country (unpublished county directorate of veterinary services reports), and this could be attributed to several factors, including low vaccination coverage and degradation of vaccine before inoculation due to cold chain unavailability and/or failures during vaccination campaigns. In addition, no study has assessed the relationship between PPR and CCPP infections in Kenya despite the infectious nature of PPR for immune cells [24] and the latent infectiousness of CCPP, a member of the *Mycoplasma* cluster.

The present study was prompted by an outbreak of an unknown respiratory disease in Kwale County, Kenya, in which the treatment response of goats in the region to antibiotics was unsatisfactory. Combined with the typical symptoms, PPR and CCPP were highly suspected. Therefore, PPR and CCPP seropositivity in goats in the four Kwale subcounties was therefore investigated. 

In this study, the disease manifestation of the sampled goats was diarrhea, frothy nasal discharges, mouth sores, and difficulty in breathing. Despite these symptoms being typical of PPR and CCPP, other diseases, including gastrointestinal helminth infestations and pneumonic pasteurellosis, present with the same symptoms [25], which underlines the importance of a definitive diagnosis. Screening for PPR Abs was performed using cELISA, while CCPP was screened using LAT. The overall PPR seropositivity was 48.6%. A previous study by Kihu et al. [26] showed that, in 2011, the seropositivity for PPR in goats in Turkana, a region with a climate similar to that of Kwale, was 55.3%. Even though the exact time of outbreak before both surveys is unknown, the seropositivity for PPR in both outbreaks was very high. With a seropositivity of 48.6%, PPR could have caused morbidities close to 50% during the outbreak period. Regionally, a higher PPR seropositivity of 74.6% was reported in Loliondo, Tanzania [27]. Kwale County and Loliondo have distinct climatic conditions, an important factor that influences the time and intensity of disease outbreaks. Situated at an altitude of 0 feet above the sea level, Loliondo experiences a temperate highland tropical climate with dry winters, classified as temperate and warm summers. The average annual temperature in Loliondo is 22.93°C (73.27°F) and receives around 83.38 millimeters (3.28 inches) of rainfall per year, spread over 125.25 rainy days, accounting for approximately 34.32% of the total time. In contrast, Kwale experiences a climate characterized by hot and overcast summers, while the winters are dry, windy, and mostly clear. The weather in Kwale can be described as oppressive throughout the year. In terms of temperature, the average range varies from 67°F to 89°F, with rare occurrences of temperatures falling below 65°F or exceeding 91°F. The climate is one important factor influencing the probability of exposure of goats to diseases [28]. A PPR prevalence of 55.3% has been reported in Uganda [29], and a very high prevalence of PPR (90.7%) was reported in Sudan in 2018 [30]. The differences in PPR prevalence could further be explained by variations in the time of sampling.

In the present study, the overall CCPP seropositivity was 45.4%. CCPP has also been reported in some East African countries, including Ethiopia (8.5%) [12] and Uganda (17.7%–23.3%) [31]. In Pakistan, CCPP seroprevalence was reported to be at 32.5% [32]. The extent of multiple infections caused by various pathogens, such as viruses, bacteria, and parasites in sheep and goats, leading to respiratory diseases, is not well-documented [33]. PPR virus damages white blood cells, inducing immunosuppression which may increase the risk or exacerbate the pathogenicity of other infections, including CCPP. In the present study, the coinfection of PPR and CCPP was found to be 22.3%. While the PPR and CCPP seropositivities in Kwale County were found to be 48.6.7% and 45.4%, respectively, there was a wide regional variation across the four subcounties. Particularly, both PPR and CCPP were highest in Kinango, which could be attributed to the more active goat trade in the subcounty, which increases goat mingling and the subsequent spread of the disease. A previous study revealed no association between the PPR virus and environmental factors such as stormy weather and strong winds [34]. However, uncontrolled animal movement and nutritional or climatic stressors increased the susceptibility of goats to CCPP [14]. Further analyses revealed no significant difference in PPR seropositivity among kids, weaners, and mature goats, and this is consistent with the separate findings in Sudan [35], Nepal [34], India [36], and Bangladesh [37]. Similarly, no significant difference was found in CCPP seropositivity among kids, weaners, and mature goats, and this is consistent with the previous findings from Pakistan [32] and Tanzania [38]. Conversely, a related study in Nigeria [39] revealed a higher PPR seropositivity in young goats. The reason for this discrepancy is not clear, but it may be related to underlying infections, nutrition, stress, and even climate, which may be harsher to kids than to mature goats.

The present study showed no significant difference in PPR and CCPP seropositivity between male and female goats. A higher CCPP seropositivity in females has, however, been reported in Tanzania [38], and this might be because female goats are kept longer for reproduction [12]; their immunity may weaken as they get older, thus increasing their risk of diseases. Univariate analysis revealed no relationship between PPR and CCPP infection despite a 22.3% PPR-CCPP coinfection, and this might be because the two diseases are endemic in the region and the exposure risks are comparable. Despite the lack of association, a high seropositivity implies that mass vaccination campaigns against PPR and CCPP in Kwale County are needed.

The findings of the current study will provide an insight into PPR and CCPP prevalence in Kwale County, which is one of the country's pastoral regions. Data on PPR are particularly important; it will enable the formulation of measures aligned with the disease eradication plan by 2030. Also, the findings of this research will create a rationale for making sound decisions in establishing specific control strategies against the diseases, with the primary aim of improving goat productivity in endemic areas. For the purpose of comprehending the evolution of the PPRV and Mccp isolates circulating in Kwale County, further studies, such as the characterization of these pathogens, are needed. Also, vaccines that induce antibodies that are unique from natural exposure are needed for accurate monitoring of disease cases; at present, surveys tend to rely on the memory of the farmers, which may be inaccurate.

## 6. Conclusion

Although univariate analysis revealed no relationship between PPR and CCPP, the rate of coinfections with the two diseases was high in Kwale County. However, given the high exposure rates to the two diseases, as a result of separate or coinfection, there is a need to upscale/intensify vaccination in the county. Future studies should focus on characterizing PPR virus isolates to identify the strains circulating in the county.

## Figures and Tables

**Table 1:** Seroprevalence of PPR and CCPP in goats in Kwale subcounties.

Subcounties	PPR-ELISA positive reaction (%)	CCPP-LAT positive reaction (%)
Kinango	65 (51.6%)	89 (70.6%)
Lunga Lunga	53 (49.1%)	32 (29.6%)
Matuga	26 (36.6%)	35 (49.3%)
Msambweni	23 (36.5%)	23 (36.6%)
Overall positivity	167 (45.4%)	178 (48.6%)

**Table 2:** The overall prevalence of PPR, CCPP, and PPR-CCPP in Kwale County.

			CCPP-LAT		Total
			Negative	Positive	
PPR-ELISA	Negative	Count	104	85	189
		% within PPR-ELISA	55.0%	45.0%	100.0%
		% within CCPP-LAT	51.7%	50.9%	51.4%
		% of total	28.3%	23.1%	51.4%
	Positive	Count	97	82	179
		% within PPR-ELISA	54.2%	45.8%	100.0%
		% within CCPP-LAT	48.3%	49.1%	48.6%
		% of total	26.4%	22.3%	48.6%

Total		Count	201	167	368
		% within PPR-ELISA	54.6%	45.4%	100.0%

**Table 3:** Relationship between sex and age of the goat and PPR infection.

	Negative *n*(%)	Positive *n*(%)	Crude odds ratio	(95% conf. interval)		*P*
*Sex*						
F	138 (53.28)	121 (46.72)	Ref			
M	51 (46.79)	58 (53.21)	1.297035	0.8282794	2.031076	0.256

*Age*						
A	113 (55.12)	92 (44.88)	Ref			
K	23 (44.23)	29 (55.77)	1.548677	0.8393203	2.85755	0.162
W	53 (47.75)	58 (52.25)	1.344135	0.8459029	2.135822	0.211

F, female goat; M, male goat; A, adult goat; K, kid (young one of a goat); W, weaner goat.

**Table 4:** Relationship between sex as well as the age of the goat and CCPP infection.

	Negative *n*(%)	Positive *n*(%)	Crude odds ratio	95% confidence interval		*P* value
*Gender*						
F	140 (54.05)	119 (45.95)	Ref			
M	61 (55.96)	48 (44.04)	0.925747	0.590135	1.452224	0.737

*Age*						
A	106 (51.71)	99 (48.29)	Ref			
K	31 (59.62)	21 (40.38)	0.725318	0.390973	1.34558	0.308
W	64 (57.66)	47 (42.34)	0.786301	0.493599	1.252574	0.312

F, female; M, male; A, adult goat; K, kid (young one of a goat); W, weaner goat.

## Data Availability

The data used to support the findings of the study, particularly the PPR cELISA results, are available from the corresponding author upon request.

## Supplementary Materials

Supplementary Materials Supplementary Figure 1: agarose gel showing the size of *Mycoplasma capricolum* subsp. *capripneumoniae* (Mccp) polymerase chain reaction (PCR) products. *L* represents a 100 bp ladder (Invitrogen) and *S* represents the PCR products for the Mccp gene, which is 316 bp, loaded in duplicate (white arrows). Supplementary Figure 2: sample LAT test results for (a) positive and (b, c) negative results.
