# Supplementary material for: Seroprevalence of Peste des Petits Ruminants and Contagious Caprine Pleuropneumonia Coinfections in Goats in Kwale County, Kenya
Source: Vet Med Int. 2023 Jul 14;2023:5513916. doi: 10.1155/2023/5513916 (PMC10361832; doi:10.1155/2023/5513916)
Supplement: Supplementary Materials — Supplementary Figure 1: agarose gel showing the size of Mycoplasma capricolum subsp. capripneumoniae (Mccp) polymerase chain reaction (PCR) products. L represents a 100 bp ladder (Invitrogen) and S represents the PCR products for the Mccp gene, which is 316 bp, loaded in duplicate (white arrows). Supplementary Figure 2: sample LAT test results for (a) positive and (b, c) negative results. [file 5513916.f1.zip › Supplementary Figure 1..pptx]

## Slide 1
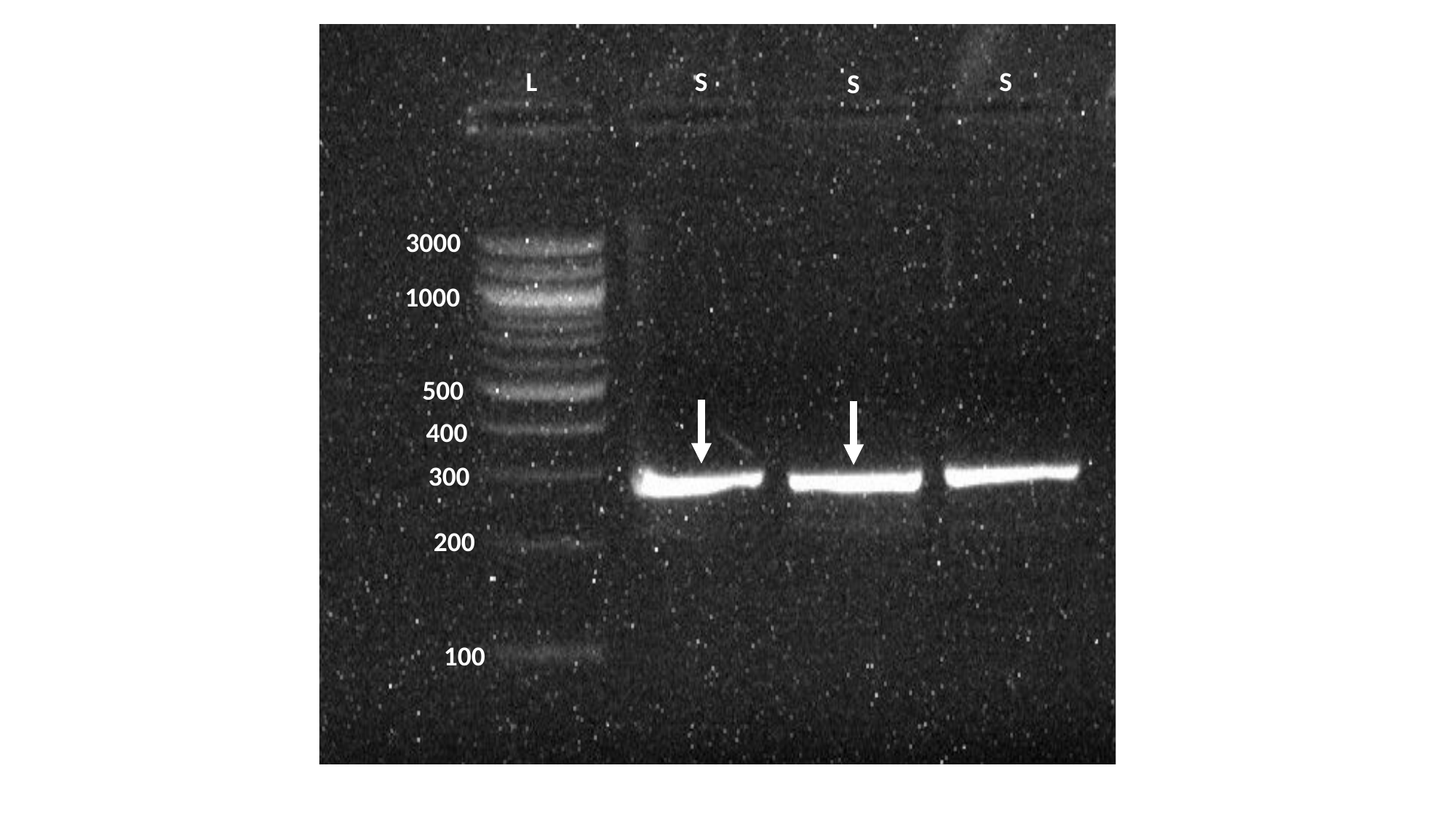

L
S
S
S
3000
1000
500
400
300
200
100

## Slide 2
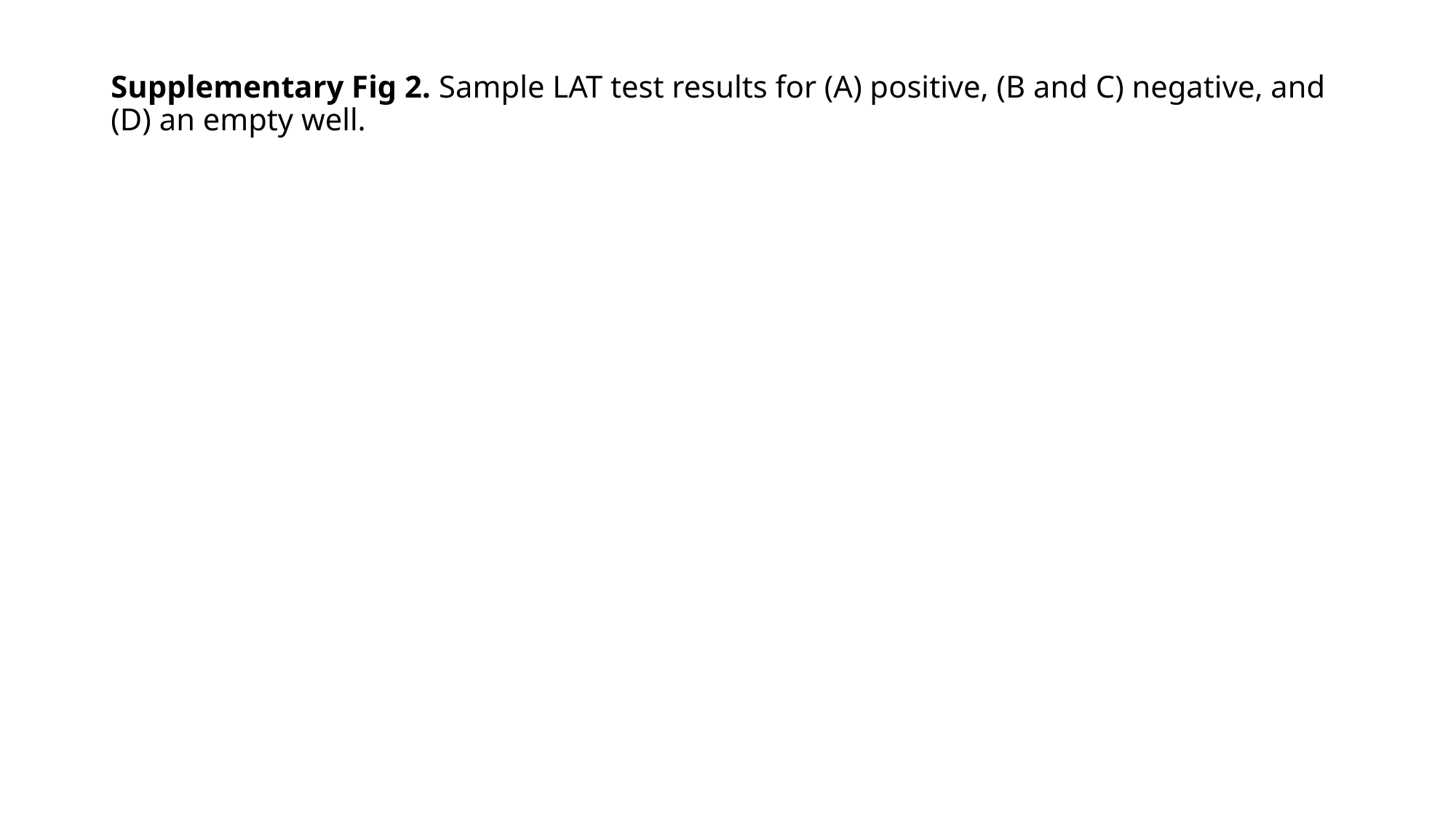

# Supplementary Fig 2. Sample LAT test results for (A) positive, (B and C) negative, and (D) an empty well.
